# Sodium scandium diphosphate, NaScP_2_O_7_, isotypic with α-NaTi(III)P_2_O_7_

**DOI:** 10.1107/S1600536809046224

**Published:** 2009-11-07

**Authors:** Jan Cempírek, Radek Škoda, Zdirad Žák

**Affiliations:** a Moravian Museum, Department of Mineralogy and Petrography, Zelný trh 6, 65937 Brno, Czech Republic; b Masaryk University, Institute of Geological Sciences, Kotlářská 2, 61137 Brno, Czech Republic; c Masaryk University, Department of Inorganic Chemistry, Kotlářská 2, 61137 Brno, Czech Republic

## Abstract

Crystals of the title compound, NaScP_2_O_7_, were grown by a flux method. The crystal structure is isotypic with those of α-NaTiP_2_O_7_, NaYbP_2_O_7_ and NaLuP_2_O_7_, and is closely related to that of NaYP_2_O_7_. The structural set-up consists of a three-dimensional framework of P_2_O_7_ units that are corner-shared by ScO_6_ octa­hedra, forming tunnels running parallel to [010]. The Na atoms are situated in the tunnels and are surrounded by nine O atoms in a distorted environment.

## Related literature

Previous X-ray powder data of NaScP_2_O_7_ were reported by Vitins *et al.* (2000 ▶). NaScP_2_O_7_ is isotypic with α-NaTiP_2_O_7_ (Leclaire *et al.*, 1988 ▶), NaYbP_2_O_7_ (Férid *et al.*, 2004 ▶) and NaLuP_2_O_7_ (Yuan *et al.*, 2007 ▶) and shows similar structural features as NaYP_2_O_7_ (Hamady & Jouini, 1996 ▶). Both structure types are topologically related to β-cristobalite (Leclaire *et al.*, 1988 ▶). For a detailed review on the structures of *A* ^I^ *M* ^III^P_2_O_7_-type diphosphates, see: Li *et al.* (2005 ▶); Schwendtner & Kolitsch (2004 ▶). For possible applications as scintillators or phosphor materials based on *A* ^I^ *M* ^III^P_2_O_7_-type diphosphates, see: Hizhnyi *et al.* (2007 ▶, 2008 ▶). For background to structural parameters, see: Brese & O’Keeffe (1991 ▶); Robinson *et al.* (1971 ▶).

## Experimental

### 

#### Crystal data

NaScP_2_O_7_
*M* *_r_* = 241.89
Monoclinic, 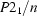

*a* = 8.9044 (18) Å
*b* = 5.3300 (11) Å
*c* = 12.516 (3) Å
β = 104.11 (3)°
*V* = 576.1 (2) Å^3^
*Z* = 4
Mo *K*α radiation
μ = 1.89 mm^−1^
*T* = 293 K
0.40 × 0.15 × 0.05 mm

#### Data collection

Kuma KM-4-CCD diffractometer
Absorption correction: multi-scan (*CrysAlis CCD*; Oxford Diffraction, 2003 ▶) *T*_min_ = 0.067, *T*_max_ = 0.093
5082 measured reflections
1018 independent reflections
932 reflections with *I* > 2σ(*I*)
*R*_int_ = 0.028

#### Refinement

*R*[*F* ^2^ > 2σ(*F* ^2^)] = 0.025
*wR*(*F* ^2^) = 0.082
*S* = 1.18
1018 reflections
101 parameters
Δρ_max_ = 0.46 e Å^−3^
Δρ_min_ = −0.46 e Å^−3^

### 

Data collection: *CrysAlis CCD* (Oxford Diffraction, 2003 ▶); cell refinement: *CrysAlis CCD*; data reduction: *CrysAlis RED* (Oxford Diffraction, 2003 ▶); program(s) used to solve structure: *SHELXS97* (Sheldrick, 2008 ▶); program(s) used to refine structure: *SHELXL97* (Sheldrick, 2008 ▶); molecular graphics: *ATOMS* (Dowty, 2003 ▶); software used to prepare material for publication: *SHELXL97*.

## Supplementary Material

Crystal structure: contains datablocks I, global. DOI: 10.1107/S1600536809046224/wm2274sup1.cif

Structure factors: contains datablocks I. DOI: 10.1107/S1600536809046224/wm2274Isup2.hkl

Additional supplementary materials:  crystallographic information; 3D view; checkCIF report

## Figures and Tables

**Table:** 

Sc—O3	2.0217 (19)
Sc—O6^i^	2.0770 (17)
Sc—O7^ii^	2.1112 (17)
Sc—O1	2.1220 (16)
Sc—O2	2.1220 (16)
Sc—O4	2.1506 (18)
P1—O6	1.5088 (17)
P1—O7	1.5254 (17)
P1—O4	1.5313 (18)
P1—O5^iii^	1.6114 (17)
P2—O3	1.5013 (19)
P2—O1^iv^	1.5278 (16)
P2—O2^v^	1.5332 (16)
P2—O5	1.6151 (17)

**Table:** 

P1^vi^—O5—P2	125.47 (10)

Symmetry codes: (i) 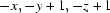; (ii) 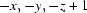; (iii) 
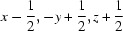
; (iv) 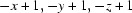; (v) 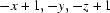; (vi) 
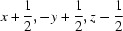
.

**Fig. 1.**
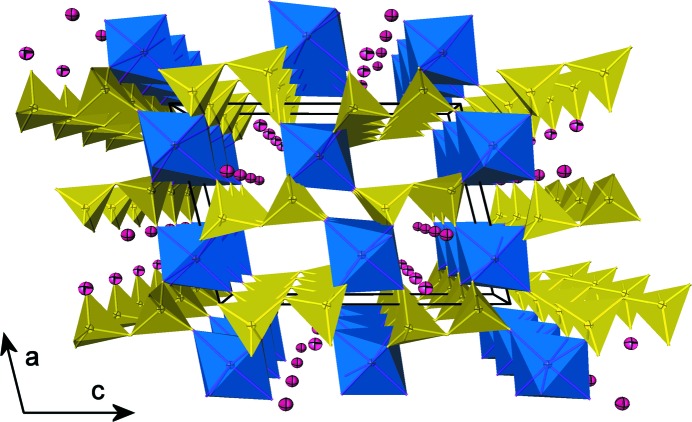
Perspective view of the NaScP2O7 framework structure projected down [010]. Diphosphate groups are corner-linked to the deformed ScO6 octahedra. Tunnels parallel to [010] are occupied by nine-coordinated atoms of Na. Displacement ellipsoids are drawn at the 50% probability level.

**Fig. 2.**
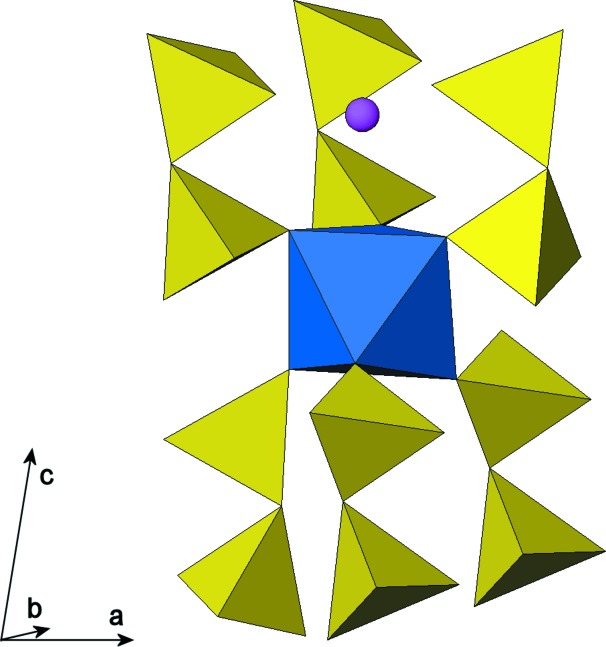
View on six P2O7 groups corner-linked to the ScO6 polyhedron.

**Fig. 3.**
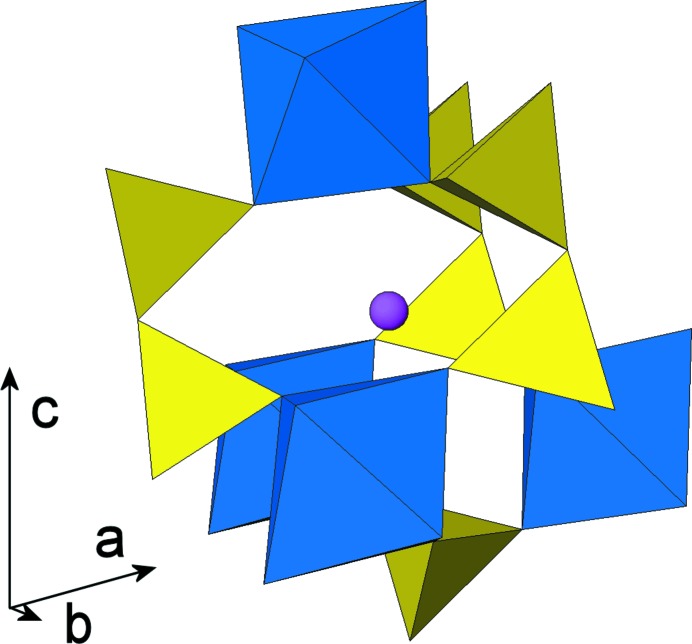
Cage formed by three diphosphate groups and four ScO6 polyhedra enclosing the Na cation.

## supplementary crystallographic information

### Comment

*A*^I^*M*^III^T^V^_2_O_7_-type compounds recently have received an increased attention, partly due to their possible applications as scintillators or phosphor materials (Hizhnyi *et al.*, 2007; Hizhnyi *et al.*, 2008). So far, the A^I^*M*^III^P_2_O_7_-type diphosphates are known to adopt eight different structure types which depends on the ratio of ionic radii of the alkali metal and the rare earth element or the three-valent metal *M*^III^. Among the eight different structure types, the KAlP_2_O_7_-type structures are most common. For a detailed review including also diarsenates, see: Schwendtner & Kolitsch (2004); Li *et al.* (2005). In this article we present the structure of NaScP_2_O_7_ determined from single-crystal *x*-ray diffaction data. Previous X-ray powder data of NaScP_2_O_7_ were reported by Vitins *et al.* (2000). However, authors could not index all reflections at that time, probably because of by-products. The crystal structure of the title compound is isotypic with α-NaTiP_2_O_7_ (Leclaire *et al.*, 1988), NaYbP_2_O_7_ (Férid *et al.*, 2004) and NaLuP_2_O_7_ (Yuan *et al.*, 2007). It is also closely related to that of NaYP_2_O_7_ (Hamady and Jouini, 1996) and β-cristobalite (Leclaire *et al.*, 1988).

All atoms in the crystal structure occupy general positions. The structure is characterized by a three-dimensional framework of PO_4_ tetrahedra (forming P_2_O_7_ groups via corner-sharing) and ScO_6_ octahedra leading to narrow tunnels parallel to [010] which are occupied by Na atoms (Fig. 1). One ScO_6_ octahedron is corner-linked to six tetrahedra of six different diphosphate groups, which are all oriented approximately perpendicular to (001) (Fig. 2). Tunnels are formed by stacking pseudohexagonal rings of [Sc_2_P_4_O_22_] units. A cage enclosing one Na atom is formed by three P_2_O_7_ groups, connected to four ScO_6_ octahedra (Fig. 3).

The P—O bond-lengths range between 1.5088 (17) Å and 1.5332 (16) Å for terminal O of the diphosphate group that are connected to octahedra. The P1—O5_bridge_—P2 angle is 125.47 (10) °, and corresponding bond lengths to the bridging O atom are 1.6114 (17) Å and 1.6151 (17) Å for <P1—O5> and <P2—O5>, respectively. The average Sc—O bond length is 2.101 Å, corresponding well with the average value for oxide compounds (2.105 Å; Brese & O'Keeffe, 1991). The ScO_6_ octahedron is significantly less distorted (in terms of quadatic elongation; Robinson *et al.*, 1971) in comparison with the equivalent polyhedra in α-NaTi^3+^P_2_O_7_, NaLuP_2_O_7_ and NaYP_2_O_7_; the polyhedral distortion is the lowest in NaYbP_2_O_7_ structure.

### Experimental

NaScP_2_O_7_ crystals were grown by the flux-growth technique. The flux, sodium hexametaphosphate (NaPO_3_)_6_ (purity 3 N) was mixed together with Sc_2_O_3_ (purity 4 N) at a molar ratio of 6:1. The mixture was filled into a platinum crucible, covered by a loose fitting lid, and heated up to 1593 K within 3 h. The temperature was held for 24 h and afterwards slowly cooled down to 1503 K in the course of 72 h. The solidified flux was dissolved in hot water and crystals of NaScP_2_O_7_ were mechanically separated. The procedure produced transparent to translucent, colorless skeletal aggregates of tabular to acicular crystals, up to 23 mm in lengths. A fragment of a crystal was used for single-crystal structure determination.

### Crystal data

**Table:**

NaScP_2_O_7_	*F*(000) = 472
*M**_r_* = 241.89	*D*_x_ = 2.789 Mg m^−^^3^
Monoclinic, *P*2_1_/*n*	Mo *K*α radiation, λ = 0.71073 Å
Hall symbol: -P 2yn	Cell parameters from 5348 reflections
*a* = 8.9044 (18) Å	θ = 4.2–27.2°
*b* = 5.3300 (11) Å	µ = 1.89 mm^−^^1^
*c* = 12.516 (3) Å	*T* = 293 K
β = 104.11 (3)°	Platy to fibrous fragment, colourless
*V* = 576.1 (2) Å^3^	0.40 × 0.15 × 0.05 mm
*Z* = 4	

### Data collection

**Table:**

Kuma KM-4-CCD diffractometer	1018 independent reflections
Radiation source: fine-focus sealed tube	932 reflections with *I* > 2σ(*I*)
graphite	*R*_int_ = 0.028
Detector resolution: 0.06 pixels mm^-1^	θ_max_ = 25.0°, θ_min_ = 4.2°
ω scans	*h* = −10→10
Absorption correction: multi-scan (*CrysAlis CCD*; Oxford Diffraction, 2003)	*k* = −4→6
*T*_min_ = 0.067, *T*_max_ = 0.093	*l* = −14→14
5082 measured reflections	

### Refinement

**Table:**

Refinement on *F*^2^	Primary atom site location: structure-invariant direct methods
Least-squares matrix: full	Secondary atom site location: difference Fourier map
*R*[*F*^2^ > 2σ(*F*^2^)] = 0.025	*w* = 1/[σ^2^(*F*_o_^2^) + (0.0535*P*)^2^ + 0.089*P*] where *P* = (*F*_o_^2^ + 2*F*_c_^2^)/3
*wR*(*F*^2^) = 0.082	(Δ/σ)_max_ < 0.001
*S* = 1.18	Δρ_max_ = 0.46 e Å^−^^3^
1018 reflections	Δρ_min_ = −0.46 e Å^−^^3^
101 parameters	Extinction correction: *SHELXL97* (Sheldrick, 2008), Fc^*^=kFc[1+0.001xFc^2^λ^3^/sin(2θ)]^-1/4^
0 restraints	Extinction coefficient: 0.080 (5)

### Special details

**Table:**

Geometry. All e.s.d.'s (except the e.s.d. in the dihedral angle between two l.s. planes) are estimated using the full covariance matrix. The cell e.s.d.'s are taken into account individually in the estimation of e.s.d.'s in distances, angles and torsion angles; correlations between e.s.d.'s in cell parameters are only used when they are defined by crystal symmetry. An approximate (isotropic) treatment of cell e.s.d.'s is used for estimating e.s.d.'s involving l.s. planes.
Refinement. Refinement of *F*^2^ against ALL reflections. The weighted *R*-factor *wR* and goodness of fit *S* are based on *F*^2^, conventional *R*-factors *R* are based on *F*, with *F* set to zero for negative *F*^2^. The threshold expression of *F*^2^ > σ(*F*^2^) is used only for calculating *R*-factors(gt) *etc*. and is not relevant to the choice of reflections for refinement. *R*-factors based on *F*^2^ are statistically about twice as large as those based on *F*, and *R*- factors based on ALL data will be even larger.

### Fractional atomic coordinates and isotropic or equivalent isotropic displacement parameters (Å^2^)

**Table:**

	*x*	*y*	*z*	*U*_iso_*/*U*_eq_	
Sc	0.26720 (5)	0.26098 (7)	0.52783 (4)	0.0137 (2)	
P1	−0.06473 (7)	0.22372 (11)	0.61678 (5)	0.0140 (2)	
P2	0.52089 (7)	0.25413 (10)	0.35283 (5)	0.0139 (2)	
Na	0.35939 (11)	0.23101 (18)	0.81018 (9)	0.0278 (3)	
O1	0.39845 (17)	0.4953 (3)	0.65350 (12)	0.0177 (4)	
O2	0.36250 (17)	−0.0381 (3)	0.63494 (13)	0.0182 (4)	
O3	0.4256 (2)	0.2456 (3)	0.43658 (14)	0.0210 (4)	
O4	0.10881 (19)	0.2727 (3)	0.63286 (14)	0.0187 (4)	
O5	0.39984 (18)	0.2187 (3)	0.23463 (13)	0.0175 (4)	
O6	−0.16444 (17)	0.4047 (3)	0.53739 (12)	0.0220 (4)	
O7	−0.10300 (18)	−0.0507 (3)	0.58783 (12)	0.0190 (4)	

### Atomic displacement parameters (Å^2^)

**Table:**

	*U*^11^	*U*^22^	*U*^33^	*U*^12^	*U*^13^	*U*^23^
Sc	0.0124 (3)	0.0152 (3)	0.0135 (3)	0.00023 (15)	0.0032 (2)	0.00014 (16)
P1	0.0126 (4)	0.0155 (4)	0.0136 (4)	−0.0001 (2)	0.0025 (3)	−0.0002 (2)
P2	0.0127 (4)	0.0156 (4)	0.0134 (4)	0.0001 (2)	0.0033 (3)	0.0003 (2)
Na	0.0270 (7)	0.0245 (7)	0.0318 (7)	−0.0010 (4)	0.0069 (5)	0.0003 (4)
O1	0.0189 (8)	0.0161 (9)	0.0173 (8)	−0.0021 (6)	0.0028 (6)	0.0000 (6)
O2	0.0190 (8)	0.0172 (9)	0.0190 (8)	0.0026 (6)	0.0055 (6)	0.0014 (7)
O3	0.0208 (8)	0.0240 (11)	0.0196 (10)	0.0009 (6)	0.0077 (7)	0.0001 (6)
O4	0.0160 (8)	0.0238 (10)	0.0175 (9)	−0.0013 (6)	0.0062 (7)	−0.0020 (6)
O5	0.0149 (9)	0.0220 (10)	0.0156 (9)	−0.0018 (6)	0.0037 (7)	0.0005 (6)
O6	0.0240 (9)	0.0188 (10)	0.0215 (8)	0.0020 (7)	0.0022 (7)	0.0026 (7)
O7	0.0205 (8)	0.0168 (9)	0.0193 (8)	−0.0009 (7)	0.0041 (6)	−0.0006 (7)

### Geometric parameters (Å, °)

**Table:**

Sc—O3	2.0217 (19)	P2—O2^v^	1.5332 (16)
Sc—O6^i^	2.0770 (17)	P2—O5	1.6151 (17)
Sc—O7^ii^	2.1112 (17)	Na—O1	2.5066 (18)
Sc—O1	2.1220 (16)	Na—O7^vi^	2.5176 (19)
Sc—O2	2.1220 (16)	Na—O4^vii^	2.5410 (19)
Sc—O4	2.1506 (18)	Na—O2^vi^	2.5597 (19)
P1—O6	1.5088 (17)	Na—O2	2.6264 (19)
P1—O7	1.5254 (17)	Na—O4	2.746 (2)
P1—O4	1.5313 (18)	Na—O1^vii^	2.7505 (19)
P1—O5^iii^	1.6114 (17)	Na—O4^vi^	2.9710 (19)
P2—O3	1.5013 (19)	Na—O6^viii^	2.992 (2)
P2—O1^iv^	1.5278 (16)		
			
O3—Sc—O6^i^	96.54 (6)	O4^vii^—Na—O2^vi^	115.31 (6)
O3—Sc—O7^ii^	93.04 (7)	O1—Na—O2	67.77 (6)
O6^i^—Sc—O7^ii^	91.19 (6)	O7^vi^—Na—O2	119.51 (6)
O3—Sc—O1	96.23 (7)	O4^vii^—Na—O2	71.71 (6)
O6^i^—Sc—O1	84.07 (7)	O2^vi^—Na—O2	130.74 (5)
O7^ii^—Sc—O1	170.01 (6)	O1—Na—O4	64.06 (6)
O3—Sc—O2	95.73 (6)	O7^vi^—Na—O4	143.35 (6)
O6^i^—Sc—O2	164.29 (6)	O4^vii^—Na—O4	108.52 (6)
O7^ii^—Sc—O2	97.93 (7)	O2^vi^—Na—O4	69.49 (6)
O1—Sc—O2	84.87 (7)	O2—Na—O4	62.69 (6)
O3—Sc—O4	176.82 (7)	O1—Na—O1^vii^	131.81 (5)
O6^i^—Sc—O4	85.62 (6)	O7^vi^—Na—O1^vii^	141.08 (7)
O7^ii^—Sc—O4	89.25 (6)	O4^vii^—Na—O1^vii^	63.57 (5)
O1—Sc—O4	81.63 (6)	O2^vi^—Na—O1^vii^	56.31 (6)
O2—Sc—O4	81.75 (6)	O2—Na—O1^vii^	93.88 (6)
O6—P1—O7	113.28 (9)	O4—Na—O1^vii^	67.92 (5)
O6—P1—O4	112.98 (9)	O1—Na—O4^vi^	67.56 (5)
O7—P1—O4	110.77 (9)	O7^vi^—Na—O4^vi^	53.79 (5)
O6—P1—O5^iii^	105.42 (9)	O4^vii^—Na—O4^vi^	150.38 (8)
O7—P1—O5^iii^	108.54 (9)	O2^vi^—Na—O4^vi^	60.19 (5)
O4—P1—O5^iii^	105.29 (10)	O2—Na—O4^vi^	135.34 (6)
O3—P2—O1^iv^	114.55 (9)	O4—Na—O4^vi^	97.26 (6)
O3—P2—O2^v^	113.07 (9)	O1^vii^—Na—O4^vi^	116.03 (6)
O1^iv^—P2—O2^v^	110.27 (9)	O1—Na—O6^viii^	159.25 (6)
O3—P2—O5	105.75 (10)	O7^vi^—Na—O6^viii^	83.21 (6)
O1^iv^—P2—O5	105.78 (9)	O4^vii^—Na—O6^viii^	61.94 (5)
O2^v^—P2—O5	106.74 (9)	O2^vi^—Na—O6^viii^	67.85 (6)
O1—Na—O7^vi^	82.54 (6)	O2—Na—O6^viii^	132.80 (6)
O1—Na—O4^vii^	137.09 (7)	O4—Na—O6^viii^	123.78 (6)
O7^vi^—Na—O4^vii^	106.18 (6)	O1^vii^—Na—O6^viii^	58.46 (5)
O1—Na—O2^vi^	101.72 (6)	O4^vi^—Na—O6^viii^	91.81 (5)
O7^vi^—Na—O2^vi^	105.53 (6)	P1^ix^—O5—P2	125.47 (10)

Symmetry codes: (i) −*x*, −*y*+1, −*z*+1; (ii) −*x*, −*y*, −*z*+1; (iii) *x*−1/2, −*y*+1/2, *z*+1/2; (iv) −*x*+1, −*y*+1, −*z*+1; (v) −*x*+1, −*y*, −*z*+1; (vi) −*x*+1/2, *y*+1/2, −*z*+3/2; (vii) −*x*+1/2, *y*−1/2, −*z*+3/2; (viii) *x*+1/2, −*y*+1/2, *z*+1/2; (ix) *x*+1/2, −*y*+1/2, *z*−1/2.
